# SARS-CoV-2 Infection-Induced Necrotising Sarcoid Granulomatosis

**DOI:** 10.31138/mjr.20230731.si

**Published:** 2023-07-31

**Authors:** Aysu Sinem Koc, Neslihan Fener, Senol Kobak

**Affiliations:** 1 Department of Pulmonology, Istinye University Faculty of Medicine, LIV Hospital, Istanbul, Turkey,; 2 Department of Pathology, University of Health Sciences, Yedikule Chest Diseases and Thoracic Surgery Training and Research Hospital, Istanbul, Turkey,; 3 Department of Internal Medicine and Rheumatology Istinye University Faculty of Medicine, LIV Hospital, WASOG Sarcoidosis Clinic, Istanbul, Turkey

**Keywords:** SARS-CoV-2 infection, necrotising sarcoid granulomatosis, granulomatosis with polyangiitis, mimicking

## Abstract

SARS-CoV-2 infection is a pandemic that affects predominantly upper airways and lungs. It may lead to reactivation of known inflammatory rheumatic diseases and/or initiation of various granulomatous disorders. Necrotising sarcoid granulomatosis (NSG) is a rare condition that can be confused with malignancy, granulomatosis with polyangiitis, and sarcoidosis. Herein we reported the development of NSG following a SARS-CoV-2 infection which mimicked granulomatosis with polyangiitis.

## INTRODUCTION

Necrotising sarcoid granulomatosis (NSG) is a rare systemic condition characterised by sarcoid-like granulomas, granulomatous vasculitis, and varying degrees of infarct-like necrosis. It was first described in 1973, and it has been unclear since then whether it is a different entity or a spectrum of a known disease such as sarcoidosis or granulomatosis with polyangiitis (GPA).^1,2^ The most frequent symptoms are cough, fever, dyspnoea, and chest pain. Extra-pulmonary involvement is found in one third of the cases, with ocular being the most common. At imaging, multiple nodules or a solitary mass are found accompanied by mediastinal lymphadenopathy at one third of the cases. It can be clinically mistaken for GPA or malignancy on radiological examination; therefore, biopsy and histopathological evaluation are necessary. SARS CoV-2 infection is a pandemic characterised with upper airway and lung involvement that in some patients may progress to respiratory and multiorgan failure. The clinical manifestations of disease are heterogeneous, which include an asymptomatic carrier, acute respiratory disease (fever, sore throat, cough), and pneumonia of varying degrees of severity. SARS-CoV-2 may lead to reactivation and/or initiation of various inflammatory rheumatic and granulomatous diseases such as sarcoidosis and/or GPA.^3,4^ There is no data in the literature yet regarding relationship between SARS-CoV-2 infection and NSG. Herein we reported a 27-year-old male patient who developed SARS-CoV-2-induced NSG.

## CASE PRESENTATION

A 27-year-old non-smoker male patient presented to the pulmonology outpatient clinic with complaints of flu-like symptoms and dry cough starting two weeks ago. Patient’s history was obtained; lifelong never smoker and reported no medicine/illicit drug/alcohol use. There was no allergy or systemic illness history.

**Figure 1.**
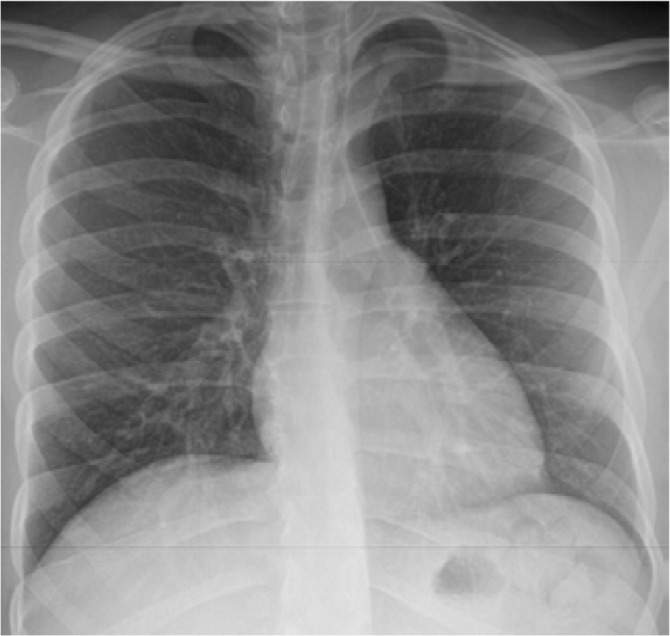
Initial lung-XR, within normal ranges.

**Figure 2.**
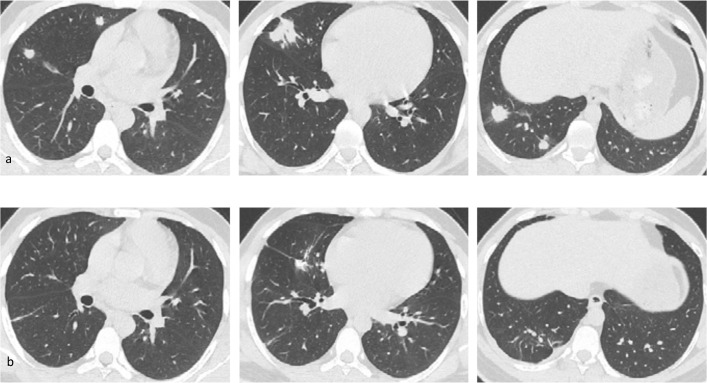
**(A)** CT scan axial images show multiple parenchymal solid nodules, largest in the right middle lobe. **(B)** Three months later, control CT images show total regression of the lesions, atelectasis due to the VATS resection.

**Figure 3.**
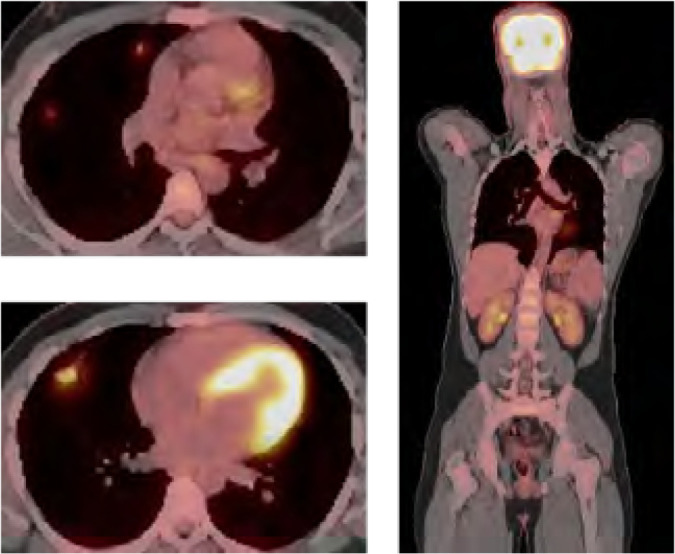
PET-CT axial and coronal images show hypermetabolic parenchymal lung nodules (SUVmax: 2,1–8,1). FDG distribution in other parts of the body is within normal limits.

**Figure 4.**
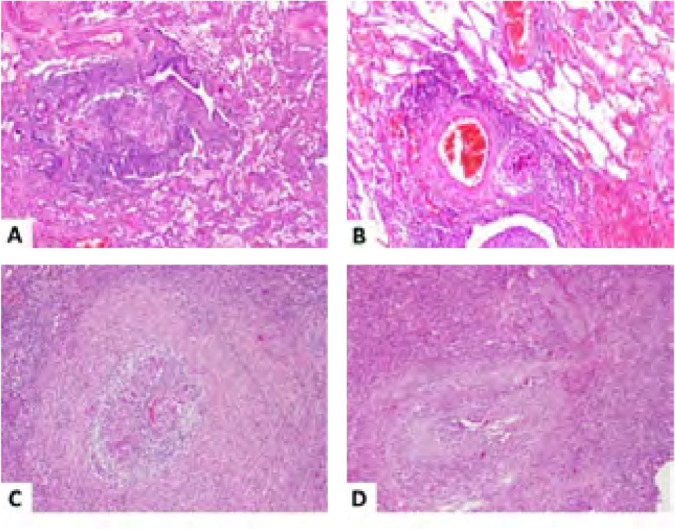
**(A)** 4x10 haematoxylin-eosin stain. Peribronchiolar non-necrotising granulomatous inflammation. **(B)** Prevascular non-necrotising granulomatous inflammation. **(C)** Necrotising sarcoid granulomatosis. The vessel wall is completely surrounded by granulomatous inflammation. The lumen of the vessel is markedly narrowed **(D)** Necrotising sarcoid granulomatosis. Lymphocyte and plasma cells can infiltrate arteries and veins. This vein wall is destroyed by the inflammatory infiltrate.

Physical examination was unremarkable. The patient works as a translator in our hospital and is at risk for SARS-CoV-2 infection; PCR test was performed and was found positive. The patient was treated only with paracetamol, no additional treatment was required. One month later, the control PCR test was negative, but a lung computed tomography (CT) was planned because he had a dry cough. The patient had a baseline normal lung-X-ray before the infection (**Figure 1**). Multiple solid nodular lesions in both lungs, with the largest measuring around 3 cm in the right lung middle lobe lateral segment were found on CT scan (**Figure 2A**). Tests were planned for the differential diagnosis of malignancy, nonspecific infections, tuberculosis, and granulomatosis with polyangiitis, based on the patient’s age and complaint profile. Laboratory tests were found as following: erythrocyte sedimentation rate (ESR): 5 mm/h (normal: 20 mm/h), C-reactive protein (CRP): 1,2 mg/dL (normal: 0–0.5 mg/dL), hydroxy-vitamin-D_3_: 6,5 ng/mL (normal: 30–80 ng/mL), angiotensin-converting enzyme (ACE): 47 U/L (normal<53 U/L). Complete blood count, serum calcium levels, liver and kidney function tests, urinalysis were found normal. Rheumatic serologic test was performed: anti-nuclear antibody, rheumatoid factor, anti-cyclic-citrullinated peptide antibody, anti-neutrophilic cytoplasmic antibodies, serum immunoglobulin levels and immunoglobulin subsets, serum complement C3/C4 levels were found to be normal. Interferon-gamma releasing assay for tuberculosis was positive, but microbiological cultures including Löwenstein-Jensen were found negative. 18-Fluorodeoxyglucose Positron emission tomography (FDG-PET) - CT was performed; apart from parenchymal nodular lesion in the lungs (standardised uptake value maximum (SUVmax range 2,1–8,1), FDG distribution was normal including hilar-mediastinal lymph nodes (**Figure 3**). The CT component of the PET confirmed that the paranasal sinuses were also normal. Bronchoscopy was scheduled for the lesion in the middle lobe, bronchoalveolar lavage and bronchoscopic transbronchial punch biopsies were performed. Histopathological findings resulted as chronic inflammation of unknown aetiology and transthoracic fine-needle aspiration biopsy (FNAB) was planned. The two consecutive biopsies resulted as benign cytology. A definitive diagnosis could not be reached after bronchoscopy and fine needle aspiration biopsy (FNAB), and video-assisted thoracoscopic surgery (VATS) biopsy was recommended, and pathology revealed between areas of massive fibrinoid necrosis, histological staining revealed clustered sarcoid granulomas and vasculitic involvement, indicating the diagnosis of NSG (**Figure 4**).

The patient was treated with 1 mg/kg/day methylprednisolone with isoniazid prophylaxis; the dose of corticosteroid then decreased gradually to 8mg/day. At month three the clinical and radiologic findings have nearly completely disappeared (**Figure 2B**). Outpatient follow-up of the patient, whose general condition is good, continues.

## DISCUSSION

SARS-CoV-2 is an infection characterised with predominantly upper airways and lungs involvement. It is a heterogenous disease which may lead to rapid acute respiratory distress syndrome (ARDS) and pulmonary insufficiency resulting in high morbidity and mortality.^5^ Immune system dysregulation and possible autoinflammatory and autoimmune mechanisms are responsible for the release of higher amounts of cytokines from immune cells, and severe clinical picture develops as a result of the “perfect cytokine storm”.^6^ As it results, SARS-CoV-2 infection may trigger many inflammatory and autoimmune disorders including granulomatous lung diseases. There are some reports in the literature about the development of granulomatous lung diseases following SARS-CoV-2 infection. Mertz et al. reported three patients with granulomatous manifestations associated with SARSCoV-2 infection.^7^ While one of these patients developed sarcoidosis, the other one had activation of established sarcoidosis disease. Capaccione et al. reported the development of sarcoid granuloma after SARS-CoV-2 infection.^8^ Our recent case is in concordance with those previously reported in the literature. Herein, we report the patient diagnosed with NSG due to SARS-CoV-2 infection which mimics GPA on radiologic evaluation. The patient’s radiologic and clinical features start after SARSCoV-2 infection, and he had no complaints previously. Wide differential diagnoses including GPA, sarcoidosis and malignancy were made and histopathological examination was compatible with NSG (**Table 1**).

**Table 1.** Comparison between main diseases causing granulomatous inflammation.

**Features**	**NSG**	**GPA**	**Sarcoidosis**	**Tuberculosis**	**Systemic mycoses**
Age (years)	8–68	40–65	Incidence peak at 25–40 Second peak in women older than 50 years(late-onset)	All age groups	All age groups
Gender	Female predominance	Female/Male equal	Female predominance	Male predominance	Female/Male equal
Race	Caucasians	Caucasians	African-American and northern European individuals	African-American, Caucasians	Asians, African-American, Caucasians
Usual presentation	Cough, fever, dyspnoea, chest pain, weight loss	Painful or painless oral ulcers, purulent or bloody nasal discharge, cough, fever, fatigue	Asymptomatic Cough, fatigue, dyspnoea in later stages of the disease fever, chills, night sweats, loss of appetite, weight loss, and fatigue	Asthma-like symptoms, fatigue, muscle aches or joint pain, night sweats, weight loss, chest pain.	
Extrapulmonary involvement	20%–30% of the patients eyes, skin, neurologic, liver, gastrointestinal tract, nose, spleen, kidney	Kidney, upper airway, trachea, skin, arthritis,	30%–70% of the patients skin, eyes, musculoskeletal system, neurologic, hearth, bones, kidney	In 15–20% of active cases; pleura, the central nervous system, the lymphatic system, the genitourinary system, and the bones and joints	blood, heart, brain, eyes, bones, skin
İmaging	Multiple nodules or solitary nodule/mass, with or without hilar lymphadenopathy	Nodules, infiltrates, cavities	Bilateral hilar lymphadenopathy, diffuse micronodular pulmonary infiltration, alveolitis, fibrosis	Infiltrate or consolidation, cavitary lesion, nodule with poorly defined margins	parenchymal nodules or consolidation with a surrounding area of ground-glass opacity(“halo”)
Pathology	Sarcoid-like granulomas Large areas of infarct-like necrosis Granulomatous vasculitis Inclusions: extremely rarely seen	Necrotising vasculitis with accompanying infiltrating cells, like neutrophils Irregularly shaped ‘dirty’ necrosis Suppurative granulomas	Non-Necrotising granulomas Small foci of central to the granulomas fibrinoid necrosis Granulomatous vasculitis with no intimal involvement Inclusions: asteroid bodies Schaumann bodies, Calcium oxalate crystals, Hamazaki-Wesenberg bodies	Multiple coalescing Necrotising granulomas with amorphous character	mixed suppurative and granulomatous reaction with varying degree of necrosis
Treatment	CS, other IS	CS, RTX, CyP	CS, other IS, TNFi	Antibiotics	Antifungal

CS: corticosteroids; RTX: Rituximab; CyP: Cyclophasphamide; IS: immunosupresive drugs; TNFi: tumour necrosis factor alpha inhibitors

The relationship between SARS-CoV-2 infection and development of granulomatous lung diseases are not known yet. One of the hypotheses are the model showing links between SARS-CoV-2 and granulomatous process mechanisms.^9^ SARS-CoV-2 acts by binding to angiotensin converting enzyme II (ACE II) receptors in target organs such as lung alveolar type 2 cells, leading to downregulation of the expression of ACE II, which in turn results in excessive production of Angiotensin II. This might lead to an excess of stimulation of angiotensin II receptor (ATR1) in innate immune cells, leading to a pro-inflammatory response that favours T-helper 1 polarization, which is responsible of the elaboration of IFN-γ, IL-2 and TNF-α. In this pro-inflammatory environment including mucosal activated invariant T cells (MAITc) activation, both ACE upregulation and/or Angiotensin II accumulation could therefore stimulate granuloma formation and lead to granulomatous responses.^10^ Therefore, SARS-CoV-2 might trigger granulomatous diseases via the renin-angiotensin system and activation of innate immune system. Some authors suggest that this MAIT cells might have protective effects from severe or life-threatening infection. These patients have less clinical symptoms and organ involvement.^11^ Recently, the efficacy of various anti-rheumatic drugs in the treatment of SARS-CoV-2 infection were reported. Cherian JJ et al. review the efficacy and safety baricitinib and tocilizumab among hospitalized patients with SARS-CoV-2 infection.^12^ They reported that baricitinib and tocilizumab both are recommended interchangeably by various guidelines for the management of SARS-CoV-2 infection considering the mortality data and other comparable efficacy and safety outcomes. Pelechas E et al. reported five patients with RA under treatment with conventional and biological DMARDs which have SARS-CoV-2 infection.^13^ The authors hypothesise that anti-rheumatic drugs may ameliorate the clinical picture and disease course of COVID-19 in RA patients. The efficacy of various DMARDs with different mechanism of action in the treatment of SARSCoV-2 infection may explain with the fact that immune system dysregulation and release of higher amounts of cytokines are responsible in its pathogenesis. Targeting the MAIT cells could be an interesting field in both SARSCoV-2 and granulomatous diseases treatment strategies. More studies in this regard are needed.

## CONCLUSION

NSG is a rare disease that can affect anyone at any age and has a wide range of differential diagnoses, the most common of which are malignancy and granulomatosis with polyangiitis. Several triggering factors were reported, and SARS-CoV-2 infection could be one of these.
